# PD223 Early Dialogue With Researchers: The Case Of The OPTIBIO Study, Innovating From The Investigation Stages

**DOI:** 10.1017/S0266462324004422

**Published:** 2025-01-07

**Authors:** Lucinda Paz-Valiñas, Sandra Rotea-Salvo, Maria del Carmen Maceira-Rozas, Beatriz Casal-Acción, María-José Faraldo Vallés

## Abstract

**Introduction:**

An early dialogue (ED) is non-binding scientific advice given to industry in the initial stages of technology development to help create evidence that the health technology assessment (HTA) agency will request. ED could also be used in the academic ecosystem. We report our experience with the clinical validation of an algorithm to predict persistent remission in patients with rheumatoid arthritis treated with biological therapy.

**Methods:**

A systematic review (SR) was undertaken to compare optimization algorithms with current clinical management. The review focused on the effectiveness and safety of these tools and included clinical practice guidelines, SRs, and primary studies. Several meetings took place between the research team and HTA researchers to integrate HTA requirements (e.g., choice of comparators, relevant outcomes, quality of life, and patient groups) into the study design to ensure the quality and accuracy assurance of data collected as well as the proper monitoring of good clinical practice.

**Results:**

Local clinical practice guidelines pointed to the importance of optimization strategies to select the most suitable patients in remission. However, there is currently no validated algorithm to select these patients. The literature search retrieved 1,809 references. There were no primary studies identified and only two ongoing randomized controlled trials met the inclusion criteria: REMRABIT-Plus (OPTIBIO) and PATIO. There were some important differences between the studies with respect to the patient populations and stages of the disease. Based on these results, the review will continue in “living evidence” mode, with the aim of collecting new evidence as it becomes available.

**Conclusions:**

There is currently an unfulfilled need between research projects in the academic context and HTA that can be resolved with ED. Collaboration in the early stages of technology development is fundamental to improving the appropriateness of data produced for future HTAs. This will facilitate quality decision-making on the incorporation of procedures into the National Health Service.

